# Multi-omics analysis reveals the alterations in the tumor microbiome and metabolome associated with cervical cancer lymph node metastasis

**DOI:** 10.1128/spectrum.02247-25

**Published:** 2026-04-30

**Authors:** Ting Zhang, Yiduo Yu, Chunyan Zhang, Mengzhuo Zhang, Shuyu Yuan, Yimeng Wang, Kaili Dai, Lijuan Zhang, Yuehui Su, Mengzhen Zhang

**Affiliations:** 1 Department of Gynecology, The First Affiliated Hospital of Zhengzhou University 824299, Zhengzhou, China; 2 Henan Key Laboratory of Cervical Cancer, The First Affiliated Hospital of Zhengzhou University 191599, Zhengzhou, China; 3 Henan Cervical Disease Prevention and Control Engineering Center, The First Affiliated Hospital of Zhengzhou University 191599, Zhengzhou, China; University of South Florida, Tampa, Florida, USA

**Keywords:** cervical cancer, lymph node metastasis, intratumor microbiota, tumor metabolism

## Abstract

Lymph node metastasis (LNM) is the primary mode of cervical cancer (CC) metastasis and is associated with a poorer prognosis. A comprehensive understanding of the underlying mechanisms driving LNM is critical to improving CC patient outcomes. Previous studies have demonstrated that alterations in the intratumor microbiome contribute to tumor progression, with metabolic regulation as a key mechanism. This study aims to investigate the role of intratumor microbiota and tumor metabolism in CC LNM. The microbiome analysis revealed significant differences in the diversity and composition of the intratumor microbiome between CC with and without LNM. *Caulobacter* and *Porphyromonas* were the most prominent genus markers for CC with LNM and CC without LNM, respectively. Alterations in tumor metabolism were also observed through global untargeted metabolomics and high-resolution spatial metabolomics, with pathways such as breast cancer, prolactin signaling, histidine metabolism, ovarian steroidogenesis, cysteine and methionine metabolism, and estrogen signaling being significantly altered in CC with LNM. Correlation analysis identified a strong association between specific intratumor microbes (especially *Porphyromonas* and *Virgibacillus*) and tumor metabolism. Additionally, our findings suggest a potential trend of phospholipid upregulation in tumor tissues during CC LNM. In summary, our findings underscore the critical role of intratumor microbiota and tumor metabolism in CC LNM, providing valuable insights for developing novel diagnostic markers and therapeutic strategies to address this challenging aspect of cervical cancer.

**IMPORTANCE:**

This study comprehensively characterized the intratumor microbiota and tumor metabolism associated with lymph node metastasis (LNM) of cervical cancer (CC) by performing 16S ribosomal RNA sequencing, global untargeted metabolomics, and high-resolution spatial metabolomics on surgical CC specimens. Our findings revealed distinct compositions and diversities of intratumor microbiota between CC with and without LNM. Each group exhibited specific bacterial genera that could potentially serve as diagnostic tools for CC LNM, and *Caulobacter* and *Porphyromonas* were the most prominent biomarkers for CC with and without LNM, respectively. The significantly different distribution trend of metabolites was also observed between CC with and without LNM. Correlation analysis identified a strong association between specific intratumor microbes (especially *Porphyromonas* and *Virgibacillus*) and tumor metabolism. This study not only provides valuable insights for developing novel diagnostic markers and therapeutic strategies for CC LNM but also contributes to a deeper understanding of the underlying molecular mechanisms of it.

## INTRODUCTION

Cervical cancer (CC) is a leading gynecological malignancy with a high incidence worldwide (1). Lymph node metastasis (LNM) significantly impacts prognosis, with patients experiencing a substantial decrease in overall survival rates (2). While the cure rate for early cervical cancer (ECC) is generally favorable, the presence of LNM drastically reduces survival (3). Consequently, lymph node status is a crucial factor in treatment planning. Despite its importance, current treatment options for CC patients with LNM remain limited and controversial. The standard surgical protocol for ECC, extensive hysterectomy plus pelvic lymph node dissection (PLND), is often performed preventively to address the potential for undetected LNM. However, given the relatively low LNM rate in ECC (15%–20%), this approach results in unnecessary PLND for a significant proportion of patients, increasing the risk of complications (4). To improve outcomes for patients with CC, a comprehensive understanding of the underlying mechanisms driving LNM is essential. This knowledge will facilitate the development of targeted biomarkers and innovative therapeutic approaches for CC with LNM. Unfortunately, our current understanding of these mechanisms remains incomplete.

The human microbiome, a complex ecosystem of microorganisms, coexists symbiotically with the human body, inhabiting all external surfaces and internal cavities. Research has implicated commensal microbes in a wide range of diseases, including mental disorders, cardiovascular diseases, and cancer (5–7). The concept of intratumor microbiota emerged in the 19^th^ century. Recent advancements in detection technology have led to the discovery of intratumor microbes in over 30 major cancer types, establishing their significance within the tumor microenvironment (8–11). While the precise origins of intratumor microbes remain unclear, potential sources include mucosal sites, adjacent tissues, and hematogenous spread (12). Intratumor microbes, primarily bacteria, are predominantly intracellular and can be found within both cancer and immune cells (11). The diversity of intratumor microbiota varies significantly across different cancer types, subtypes, and stages (13). Importantly, extensive studies have revealed that intratumor microbes can influence cancer initiation and progression through various mechanisms, including genome instability, epigenetic modifications, chronic inflammation, immune evasion, invasion and metastasis, and metabolic regulation (13).

Metabolic dysregulation is a hallmark of cancer, with metabolic phenotypes and dependencies evolving throughout tumor progression. These alterations offer potential diagnostic, prognostic, and therapeutic targets. Intratumor microbes have been demonstrated to influence cancer metabolism. For example, in breast cancer, *Fusobacterium, Atopobium, Hydrogenophaga, Gluconacetobacter,* and *Lactobacillus* can reduce inositol phosphate metabolism (14). In gastric tumors, *Helicobacter* and *Lactobacillus* play roles in amino acid, carbohydrate, nucleoside, nucleotide, and glycerophospholipid metabolism (15). *Fusobacterium nucleatum* in oral squamous cell carcinoma promotes glucose transporter 1 upregulation and lactic acid accumulation, contributing to tumor progression (16). *Escherichia coli* in colorectal cancer enhances lactate production, leading to liver metastasis (17). The microbiome’s role in CC initiation and development is well-established. *Gardnerella*, *Prevotella*, *Streptococcus*, and *Atopobium* from the vaginal microbiota have been linked to amino acid and nucleotide metabolism and CC initiation (18). *Lactobacillus iners* in CC tissue can alter tumor metabolism and lactate signaling, conferring chemotherapy and radiation resistance (19). However, the specific impact of intratumor microbes and associated metabolic alterations on cervical cancer LNM remains an area of active investigation.

This study employed 16S ribosomal RNA (rRNA) sequencing, global untargeted metabolomics, and high-resolution spatial metabolomics to analyze surgical specimens of CC with and without LNM. By comparing the microbiome and metabolic profiles between these groups and conducting a correlation analysis, we aimed to identify novel therapeutic targets and diagnostic biomarkers for CC LNM.

## MATERIALS AND METHODS

### Tissue sample collection

A total of 90 surgical specimens of CC were collected from the First Affiliated Hospital of Zhengzhou University, including 43 with LNM and 47 without LNM. Postoperative pathological examination confirmed lymph node status. The inclusion criteria for this study were as follows: patients must have a pathological confirmation of CC; no evidence of tumor metastasis was observed during preoperative examinations; patients’ preoperative staging was classified as IA1-IIA2, aligning with the National Comprehensive Cancer Network guidelines for surgical intervention; and patients must be newly diagnosed without having received any prior anti-tumor treatment. The exclusion criteria included patients with a history of smoking; a history of systemic or local anti-tumor therapies; the presence of severe comorbidities or complications that might affect study outcomes, such as significant endocrine disorders, immunocompromise, or conditions requiring long-term administration of immunosuppressants or glucocorticoids; and the tumor tissue was insufficient in size to obtain adequate samples for omics analysis without compromising the postoperative pathological diagnosis. All patients enrolled in this study underwent robot-assisted laparoscopic radical hysterectomy for CC. The samples were obtained aseptically in the operating room and subsequently washed with sterile saline to eliminate any residual blood. Then, the samples were transferred to 2 mL sterile cryotubes and stored in liquid nitrogen within 30 min from the time of sample collection.

### Immunohistochemistry

Immunohistochemistry staining was performed according to the manufacturer’s protocol. Tissue sections were incubated with primary antibodies against LPS (HycultBiotech, HM6011), CHKA (Proteintech, 13520-1-AP), CEPT1 (Proteintech, 20496-1-AP), PLD1 (Proteintech, 18355-1-AP), PLA2 (Proteintech, 22030-1-AP), and ETNK1 (CUSABIO, CSB-PA007850LA01HU), respectively. The ultraView Universal DAB Detection Kit (05269806001) was used for signal visualization.

### DNA extraction and 16S rRNA gene amplicon sequencing

Sixty surgical specimens of CC were subjected to 16S rRNA gene sequencing for bacterial community analysis. Total genomic DNA was first extracted from each sample using the OMEGA Soil DNA Kit (M5635-02) (Omega Bio-Tek, Norcross, GA, USA) following the manufacturer’s instructions. The detailed operation procedure is shown in Fig. S1. Extracted DNAs were then quantified and assessed for quality using a NanoDrop NC2000 spectrophotometer (Thermo Fisher Scientific, Waltham, MA, USA) and 1.2% agarose gel electrophoresis, respectively. Subsequently, the V3-V4 hypervariable region of the bacterial 16S rRNA gene was amplified by polymerase chain reaction using the forward primer 338F (5′-ACTCCTACGGGAGGCAGCA-3′) and the reverse primer 806R (5′-GGACTACHVGGGTWTCTAAT-3′). These primers were modified to incorporate sample-specific 7-bp barcodes for multiplex sequencing. Amplicons were purified using Vazyme VAHTS DNA Clean Beads (Vazyme, Nanjing, China) and quantified with the Quant-iT PicoGreen dsDNA Assay Kit (Invitrogen, Carlsbad, CA, USA). Following individual quantification, amplicons were pooled in equal amounts and subjected to paired-end 2 × 250 bp sequencing on the Illumina NovaSeq platform with NovaSeq 6000 SP Reagent Kit (500 cycles) at Suzhou PANOMIX Biomedical Tech Co., Ltd.

### Sequencing data analysis

Microbiome analysis was conducted using QIIME2 version 2019.4 with minor modifications based on the official tutorials (https://docs.qiime2.org/2019.4/tutorials/). Briefly, raw sequencing data underwent demultiplexing with the demux plugin, followed by primer removal using the cutadapt plugin. The DADA2 plugin then performed quality filtering, denoising, sequence merging, and chimera removal. Non-singleton amplicon sequence variants (ASVs) were aligned with MAFFT and used to construct a phylogenetic tree with FastTree2. Alpha- and beta-diversity metrics were estimated using the diversity plugin after rarefying samples to a depth of 4,941 sequences per sample. Taxonomic assignment of ASVs was achieved using the classify-sklearn naïve Bayes taxonomy classifier within the feature-classifier plugin, referencing the Greengenes Release 13.8 database. While QIIME2 facilitated the majority of sequence data analysis, R packages (version 3.2.0) were also employed. Beta diversity analysis, visualized via principal coordinate analysis (PCoA) using Jaccard metrics, investigated the structural variation of microbial communities across samples. Permutational multivariate analysis of variance within QIIME2 assessed the significance of microbiota structure differentiation among groups. Venn diagrams, generated with the R package “VennDiagram,” visualized shared and unique ASVs across samples or groups based on their occurrence, independent of relative abundance. Finally, linear discriminant analysis effect size (LEfSe) identified differentially abundant taxa between groups using default parameters.

### Metabolite extraction and global untargeted metabolomic analysis

Metabolites were extracted from the 60 surgical specimens of CC. For detailed information on sample preparation, liquid chromatography (LC)-mass spectrometry (MS) analysis, data quality control, and compound identification, please refer to the supplemental material. The LC analysis was conducted using a Vanquish UHPLC System (Thermo Fisher Scientific, USA). Chromatography was performed on an ACQUITY UPLC HSS T3 column (2.1 × 100 mm, 1.8 µm) (Waters, Milford, MA, USA). Mass spectrometric detection of metabolites was achieved using an Orbitrap Exploris 120 mass spectrometer (Thermo Fisher Scientific, USA) equipped with an electrospray ionization (ESI) ion source.

### Spatial metabolomics analysis

Six frozen tumor samples were collected and stored at −80°C for subsequent analysis. Details regarding the preparation of frozen sections, hematoxylin and eosin (H&E) staining, matrix application, and mass spectrometry conditions are provided in the supplemental material. The experiment proceeded by line-by-line scanning of the sample on a slide using an atmospheric pressure-matrix-assisted laser desorption/ionization ion source instrument. Acquired profile data from each scan line were then saved as RAW data files. High-resolution mass spectrometry imaging (MSI) image reconstruction and visualization were achieved using dedicated MSI processing algorithms. An in-house standalone software platform (http://www.biodeep.cn) provided an automated processing pipeline for the MSI data. This involved initial conversion of RAW data files into the open-source mzML format, followed by ion peak detection and integration. The threshold intensity quantization algorithm was then employed to identify background noise and adjust signal intensities for mass spectrum ion signals. Background subtraction and dimensionality reduction were performed on the resulting data. Unsupervised automatic segmentation of the MSI data set was achieved using spatial cluster analysis with the uniform manifold approximation and projection in combination with the phenograph algorithm. Regions with similar metabolite expression patterns and characteristics were assigned the same color. By combining the spatial data filling algorithm, a partition result that is highly consistent with the actual distribution characteristics of the tissue was obtained. The regions of interest were derived using the complete process script. Finally, differential metabolites were identified between spatial segments, and their biological significance was explored through Kyoto Encyclopedia of Genes and Genomes (KEGG) metabolic pathway analysis.

### Statistical analysis

Statistical analyses were performed using SPSS software (version 22.0; SPSS Inc., Chicago, IL, USA). Independent *t*-tests were employed to assess differences between two groups. Chi-square tests were conducted to compare the levels of LPS expression between cervical paracancer and cancer tissues, as well as between CC tissues with and without LNM. Pearson’s correlation analysis was used to examine the association between microbial species at the genus level and relevant metabolites, with the numerical matrix visualized through heatmaps. Statistical significance was considered at a *P*-value < 0.05.

## RESULTS

### Clinical information of participants enrolled in omics analysis

A total of 90 patients with CC provided surgical specimens for this study, comprising 43 patients with CC and lymph node metastasis (metastasis group) and 47 patients with CC without lymph node metastasis (non-metastasis group). According to the 2023 International Federation of Gynecology and Obstetrics CC staging system, all patients in the metastasis group were classified as stage IIIC CC, further divided into stage IIIC1 (29 patients) and stage IIIC2 (14 patients). In the non-metastasis group, 29 patients were diagnosed with stage IB CC, and 18 patients were diagnosed with stage IIA CC. This study involved 16S rRNA sequencing, global untargeted metabolomics, and high-resolution spatial metabolomics on 60, 60, and 6 specimens, respectively. When comparing the metastasis and non-metastasis groups in the 16S rRNA sequencing and global untargeted metabolomics analyses, there was no significant difference in age, indicating comparable age distributions between the two groups (*P* = 0.596 and *P* = 0.804) (Table 1). Detailed demographic information is provided in Table 2.

**TABLE 1:** The age analysis of participants from whom the surgical specimens were collected for 16S and UTM^*a*^

Group	Age	*P* value
16S		0.596
C	46.24 ± 10.938	
M	47.58 ± 9.462	
UTM		0.804
C	45.89 ± 10.177	
M	45.76 ± 12.411	

^*a*^ C, cervical cancer without LNM; M, cervical cancer with LNM; UTM, global untargeted metabolomics; and 16S, 16S ribosomal RNA gene amplicon sequencing.

**TABLE 2:** Clinical characteristics of participants^*a*^

Patient ID	Age	Tumor stage	Omics analysis	Patient ID	Age	Tumor stage	Omics analysis
M-1	54	IIIC1	SM	C-3	56	IIA1	SM
M-2	59	IIIC1	SM	C-4	24	IB2	16S
M-3	53	IIIC1	SM	C-5	48	IB2	16S
M-4	38	IIIC1	16S	C-6	42	IB1	16S
M-5	51	IIIC2	16S	C-7	37	IB3	16S
M-6	45	IIIC2	16S	C-8	67	IIA1	16S
M-7	51	IIIC2	16S	C-9	53	IIA1	16S
M-8	52	IIIC2	16S	C-10	40	IIA1	16S
M-9	41	IIIC1	16S	C-11	56	IB2	16S
M-10	56	IIIC1	16S	C-12	58	IB2	16S
M-11	28	IIIC1	16S	C-13	28	IB1	16S + UTM
M-12	45	IIIC2	16S	C-14	31	IB2	16S + UTM
M-13	57	IIIC1	16S	C-15	34	IB1	16S + UTM
M-14	52	IIIC1	16S	C-16	44	IIA1	16S + UTM
M-15	45	IIIC1	16S	C-17	44	IIA1	16S + UTM
M-16	56	IIIC1	16S	C-18	54	IIA1	16S + UTM
M-17	58	IIIC1	16S	C-19	54	IIA1	16S + UTM
M-18	42	IIIC2	16S	C-20	55	IIA2	16S + UTM
M-19	29	IIIC1	16S + UTM	C-21	56	IIA1	16S + UTM
M-20	33	IIIC1	16S + UTM	C-22	59	IIA1	16S + UTM
M-21	37	IIIC1	16S + UTM	C-23	59	IIA1	16S + UTM
M-22	42	IIIC2	16S + UTM	C-24	67	IB1	16S + UTM
M-23	49	IIIC1	16S + UTM	C-25	45	IB2	16S + UTM
M-24	51	IIIC1	16S + UTM	C-26	46	IB2	16S + UTM
M-25	52	IIIC1	16S + UTM	C-27	42	IB2	16S + UTM
M-26	61	IIIC1	16S + UTM	C-28	51	IB1	16S + UTM
M-27	63	IIIC1	16S + UTM	C-29	43	IB2	16S + UTM
M-28	45	IIIC2	16S + UTM	C-30	41	IB1	16S + UTM
M-29	58	IIIC2	16S + UTM	C-31	52	IB3	16S + UTM
M-30	27	IIIC1	UTM	C-32	54	IB2	16S + UTM
M-31	32	IIIC1	UTM	C-33	40	IIA1	16S + UTM
M-32	32	IIIC1	UTM	C-34	38	IB1	16S + UTM
M-33	37	IIIC1	UTM	C-35	26	IB1	16S + UTM
M-34	41	IIIC1	UTM	C-36	35	IB3	16S + UTM
M-35	41	IIIC2	UTM	C-37	49	IB2	16S + UTM
M-36	42	IIIC2	UTM	C-38	33	IB2	UTM
M-37	44	IIIC2	UTM	C-39	52	IIA1	UTM
M-38	54	IIIC1	UTM	C-40	59	IIA1	UTM
M-39	58	IIIC2	UTM	C-41	52	IB2	UTM
M-40	59	IIIC2	UTM	C-42	38	IB2	UTM
M-41	75	IIIC1	UTM	C-43	52	IB2	UTM
M-42	52	IIIC1	UTM	C-44	60	IIA1	UTM
M43	30	IIIC1	UTM	C-45	40	IB1	UTM
C-1	54	IIA1	SM	C-46	38	IB2	UTM
C-2	58	IIA1	SM	C-47	35	IB2	UTM

^*a*^ C, cervical cancer without LNM; M, cervical cancer with LNM; SM, high-resolution spatial metabolomics; UTM, global untargeted metabolomics; and 16S, 16S ribosomal RNA gene amplicon sequencing.

### Intratumoral microbial communities present in cervical cancer

**Fig 1:**
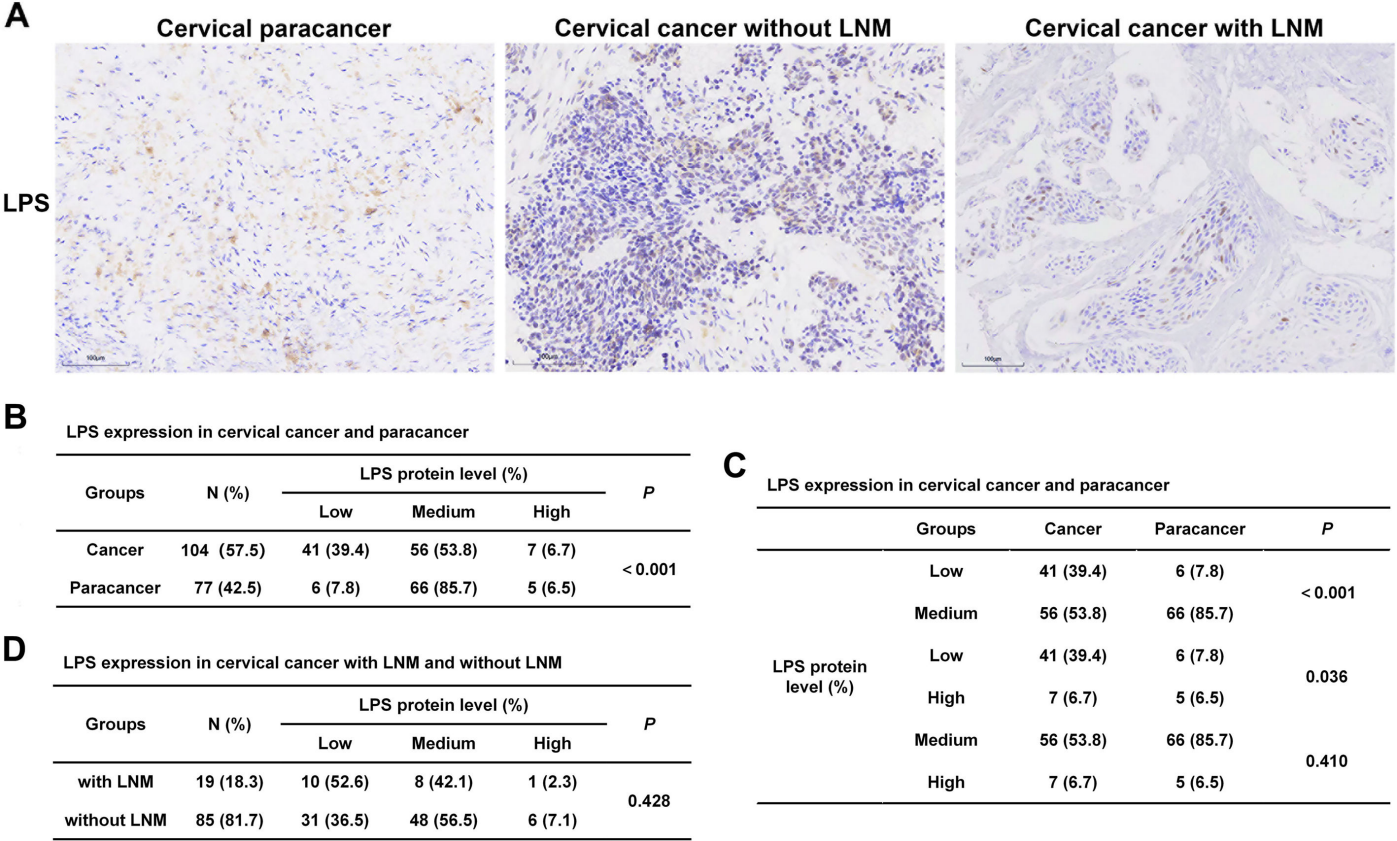
Intratumoral microbial communities present in cervical cancer. (**A–D**) LPS was expressed in cervical cancer tissues. The expression of LPS was examined by immunohistochemistry using cervical cancer tissue microarray: (**A**) representative images of LPS staining in tissues of cervical paracancer, CC without LNM, and CC with LNM. (**B–D**) Statistical analysis of differences in LPS expression in tissues of cervical paracancer, CC without LNM, and CC with LNM.

Prior to investigating the compositional and diversity disparities of intratumor microbiota between CC patients with and without LNM, we initially examined the presence of microbes in CC tissues. Immunohistochemistry (IHC) employing an LPS antibody, a marker for gram-negative bacteria, was conducted on a cervical cancer tissue microarray. The immunostaining pattern was categorized as high, medium, or low based on a scoring system evaluating total staining intensity and the proportion of positive cells. Statistical analysis revealed a significant downregulation of LPS expression in CC tissues compared to paracancer tissues (*P* < 0.001), primarily due to a higher proportion of low LPS expression in CC tissues (Fig. 1A through C). A comparison of LPS expression levels between CC tissues with and without LNM did not reveal any significant differences (Fig. 1D). Collectively, these findings demonstrate the presence of microbes within CC tissues, and the abundance of intratumor microbes does not seem to be influenced by LNM.

### Alterations in the composition and diversity of intratumor microbiota associated with LNM of CC

**Fig 2:**
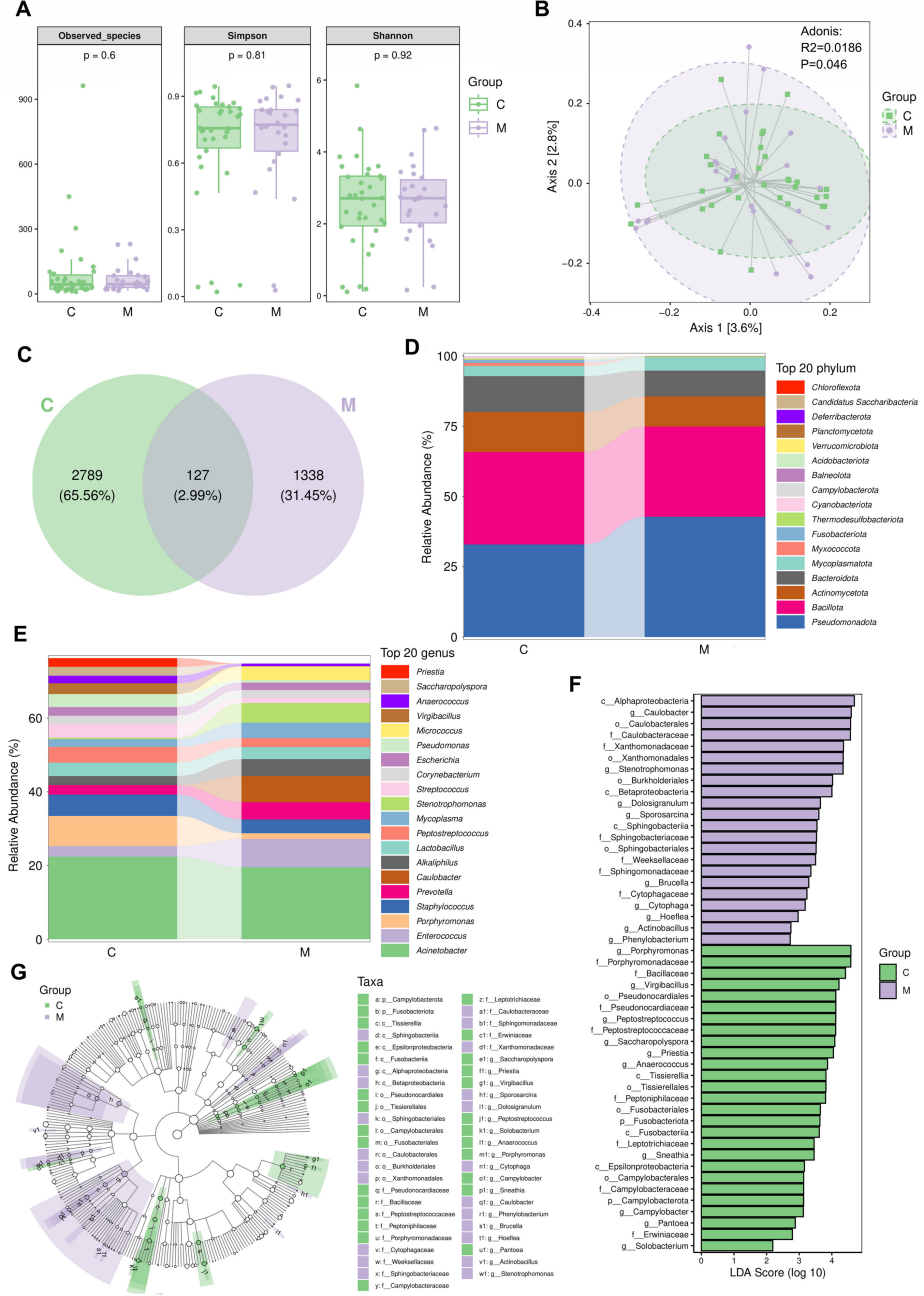
Alterations in the composition and diversity of intratumor microbiota associated with LNM of CC. (**A and B**) Analysis of the diversity of intratumor microbiota in 34 CC tissue samples without LNM (**C**) and 26 CC tissue samples with LNM (M). (**A**) Alpha diversity boxplot (observed species, Shannon, and Simpson indices) in CC without LNM and CC with LNM tissues. (**B**) PCoA using Jaccard distances of beta diversity. (**C–E**) Analysis of the composition of intratumor microbiota in 34 CC tissue samples without LNM (**C**) and 26 CC tissue samples with LNM (M). (**C**) Venn diagram showing the numbers of exclusive and shared ASV/OTU between CC without LNM and CC with LNM groups. (**D**) Taxonomic column diagram at the phylum level showing the top 20 most abundant identified phyla. (**E**) Taxonomic column diagram at genus level showing the overall top 20 most abundant identified genera. (**F and G**) Distinctive bacterial species in CC without LNM (**C**) and CC with LNM (M) groups. (**F**) Histogram of LDA score among taxa with significant differences between the two groups. (**G**) Taxonomic cladistics of LEfSe analysis.

To explore the relationship between the composition and diversity of intratumor microbiota and LNM of CC, 16S rRNA sequencing was performed on 60 surgical specimens of CC, including 26 cases with LNM and 34 cases without LNM. The alpha-diversity of the tumor microbiota was measured first. Observed species index, Simpson index, and Shannon index, which represent the within-group taxonomic richness and diversity of intratumor microbiota, were used to evaluate the alpha-diversity. It was shown that the alpha-diversities of intratumor microbiota in metastasis and non-metastasis groups were similar (Fig. 2A). Additionally, beta diversity analysis using Jaccard distances and PCoA revealed a significant difference between the metastasis and non-metastasis groups (*P* = 0.046), suggesting distinct intratumor microbial communities within each group (Fig. 2B). To further explore these compositional differences, a Venn diagram was constructed, indicating 2,789, 1,338, and 127 ASV/operational taxonomic units (OTUs) unique to the non-metastasis group, metastasis group, and shared between both groups, respectively (Fig. 2C). At the phylum level, *Pseudomonadota*, *Bacillota, Actinomycetota*, *Bacteroidota*, and *Mycoplasmatota* were the dominant phyla in both groups (Fig. 2D). However, relative to the non-metastasis group, the metastasis group exhibited increased abundances of *Pseudomonadota* and *Mycoplasmatota* while showing decreased abundances of *Actinomycetota* and *Bacteroidota* (Fig. 2D). At the genus level, *Acinetobacter* was the most dominant genus in both the metastasis and non-metastasis groups, with a slight decrease in the metastasis group compared to the non-metastasis group (Fig. 2E). Furthermore, *Porphyromonas, Staphylococcus*, and *Peptostreptococcus* were more prevalent in the non-metastasis group, whereas *Enterococcus*, *Caulobacter*, and *Stenotrophomonas* were more abundant in the metastasis group (Fig. 2E). Notably, *Enterococcus*, *Caulobacter*, *Stenotrophomonas*, *Micrococcus*, *Prevotella*, *Alkaliphilus*, and *Mycoplasma* were significantly elevated in the metastasis group compared to the non-metastasis group, while *Porphyromonas*, *Streptococcus*, *Pseudomonas*, *Virgibacillus*, *Anaerococcus, Saccharopolyspora*, *Priestia*, *Peptostreptococcus*, and *Staphylococcus* were decreased (Fig. 2E). Further LEfSe analysis identified 49 taxa with differential relative abundances between the two groups, with *Caulobacter* and *Porphyromonas* emerging as the most prominent biomarkers for the metastasis and non-metastasis groups, respectively (Fig. 2F and G). Detailed LEfSe results are provided in Table S1. Collectively, these findings highlight disparities in the composition and diversity of intratumor microbiota between CC with LNM and CC without LNM. The differential intratumor bacteria may play a role in CC LNM and could potentially serve as biomarkers for its diagnosis.

### Identification of differential metabolites between CC with and without LNM by global untargeted metabolomics

**Fig 3:**
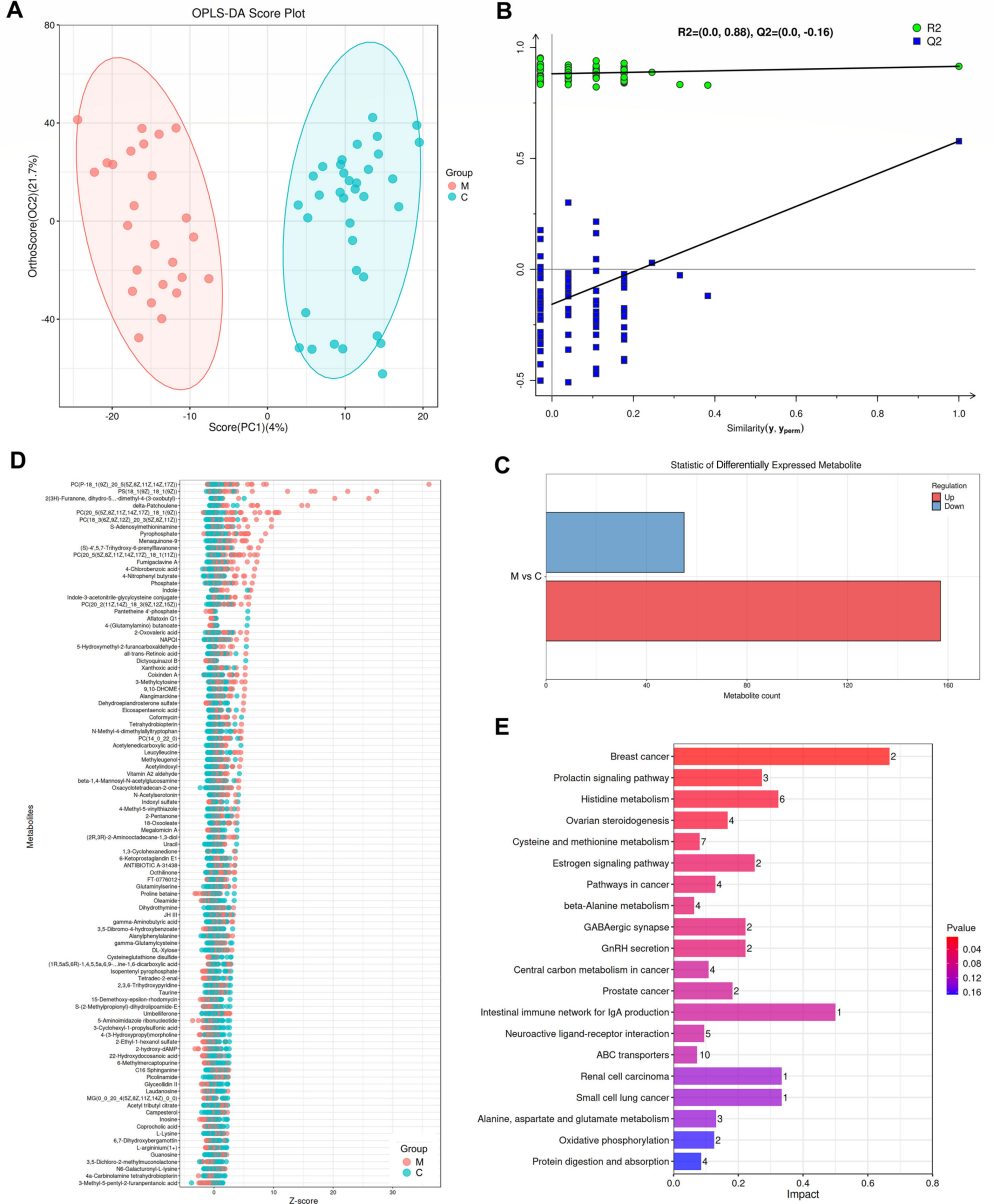
Global untargeted metabolomics reveal differential metabolites between CC with and without LNM. (**A**) Overall distribution trends of samples between CC with LNM (M, *n* = 25) and CC without LNM (C, *n* = 35) groups by the OPLS-DA. (**B**) OPLS-DA model evaluation by permutation test. (**C and D**) Differentially expressed metabolites between the two groups. (**C**) Statistical histogram of differentially expressed metabolites between the two groups (M vs C). (**D**) *Z*-score showing the overall change trend and the degree of difference of the top 100 differential metabolites. (**E**) KEGG pathway enrichment analysis of differential metabolites. The count of differential metabolites in the most enriched pathways and the impact of differential metabolites on the most enriched pathways were illustrated by a bar chart of the influence factors of the metabolic pathway. The horizontal axis shows impact values for various metabolic pathways, while the vertical axis lists these pathways. The numbers indicate the corresponding number of metabolites involved in the pathways. The color gradient reflects the *P*-values, with red hues indicating smaller *P*-values and blue hues indicating larger *P*-values.

Considering the potential involvement of intratumor microbes in tumor progression through the regulation of tumor metabolism, global untargeted metabolomics were conducted on 60 surgical specimens of CC, comprising 25 cases with LNM and 35 cases without LNM. Orthogonal Projections to Latent Structures Discriminant Analysis (OPLS-DA) was employed to visualize the distribution trends of metabolites, revealing significant differences between the metastasis and non-metastasis groups (Fig. 3A). The reliability of the OPLS-DA model was confirmed by permutation testing (Fig. 3B). As illustrated in Fig. 3C, a total of 212 metabolites were significantly differentially expressed between the two groups, with 157 metabolites upregulated and 55 metabolites downregulated in the metastasis group compared to the non-metastasis group. *Z*-scores, representing the relative metabolite content at the same level, were used to depict the overall trends and degrees of difference between the two groups. The top 100 differential metabolites are presented in Fig. 3D. To gain insights into the functional significance of these differential metabolites in CC LNM, we conducted pathway enrichment analysis using the KEGG and identified the significantly altered pathways in the metastasis group. We found that the significantly differential pathways in the metastasis group compared to the non-metastasis group were breast cancer, prolactin signaling, histidine metabolism, ovarian steroidogenesis, cysteine and methionine metabolism, and estrogen signaling (Fig. 3E). Notably, the pathways of breast cancer, prolactin signaling, ovarian steroidogenesis, and estrogen signaling were upregulated in CC with LNM (Fig. 3E). Detailed metabolite information for the two groups and the result of KEGG enrichment analysis are provided in Tables S2 and S3.

### Correlation analysis between intratumor microbiota and tumor metabolism

**Fig 4:**
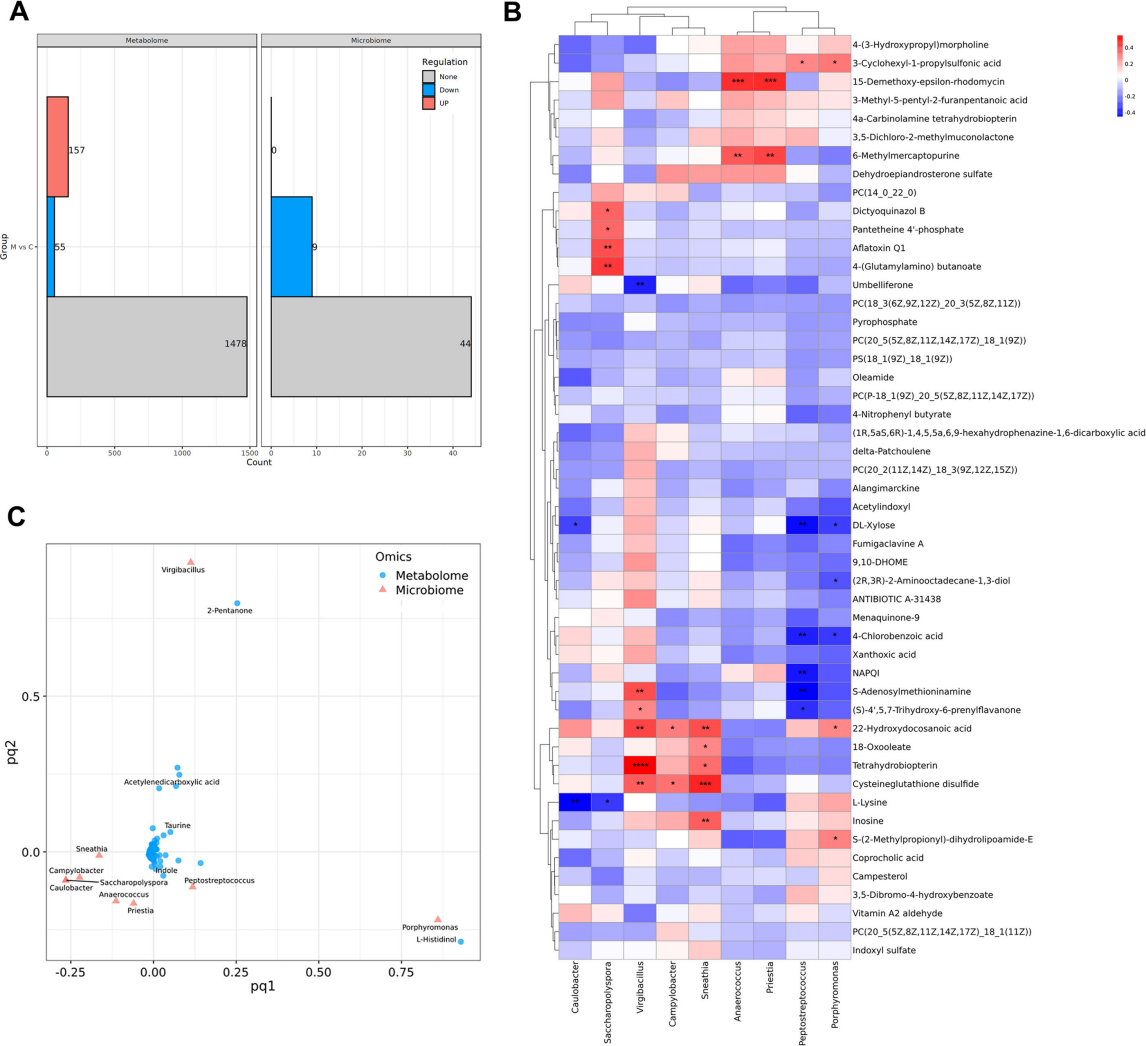
Correlation analysis between intratumor microbiota and tumor metabolism. (**A**) The histogram showing the numbers of upregulated, downregulated, and non-differentiated microbes and metabolites in CC with LNM group (M, *n* = 11) compared with CC without LNM group (C, *n* = 25). (**B**) Correlation analysis of the top 50 differential microbes and metabolites. Red represents a positive correlation, and blue represents a negative correlation. (*P < 0.05; **P < 0.01; ***P < 0.001; and ****P < 0.0001). (**C**) O2PLS analysis showing the relationship between differential microbes and differential metabolites.

To investigate the relationship between intratumor microbiota and tumor metabolism, a total of 36 samples, including 25 CC cases without LNM and 11 CC cases with LNM, were subjected to simultaneous 16S rRNA sequencing and global untargeted metabolomics analysis. As shown in Fig. 4A, the relative abundances of nine intratumor microbes differed between the CC with LNM and CC without LNM groups, with all nine microbes exhibiting downregulation in CC with LNM. In addition, compared to CC without LNM, 157 tumor metabolites were increased, and 55 were decreased in CC with LNM (Fig. 4A). Correlation analysis between the nine differential intratumor microbes and the top 50 differential metabolites revealed that the levels of cysteine-glutathione disulfide, tetrahydrobiopterin, 22-hydroxydocosanoic acid, 15-demethoxy-epsilon-rhodomycin, 6-methylmercaptopurine, and DL-xylose were most significantly affected by the differential intratumor microbes. Cysteine-glutathione disulfide and tetrahydrobiopterin levels were primarily positively regulated by *Virgibacillus* and *Sneathia*. The level of 22-hydroxydocosanoic acid was positively correlated with the relative abundances of *Virgibacillus*, *Sneathia*, *Campylobacter,* and *Porphyromonas*. The levels of 15-demethoxy-epsilon-rhodomycin and 6-methylmercaptopurine were positively regulated by *Anaerococcus* and *Priestia*. Conversely, the level of DL-xylose was negatively correlated with the relative abundances of *Caulobacter, Peptostreptococcus*, and *Porphyromonas* (Fig. 4B). In addition, although not statistically significant, negative correlations were observed between the levels of PC[18:3(6Z,9Z,12Z)/20:3(5Z,8Z,11Z)], PC[20:5(5Z,8Z,11Z,14Z,17Z)/18:1(9Z)], PS[18:1(9Z)/18:1(9Z)], and PC[P-18:1(9Z)/20:5(5Z,8Z,11Z,14Z,17Z)] and the relative abundance of these intratumor microbes (Fig. 4B). Two-way Orthogonal Partial Least Squares (O2PLS) analysis further indicated that the relative abundances of *Porphyromonas* and *Virgibacillus* exerted the greatest influence on tumor metabolites (Fig. 4C).

### Characterization of the metabolome in CC by high-resolution spatial metabolomics

**Fig 5:**
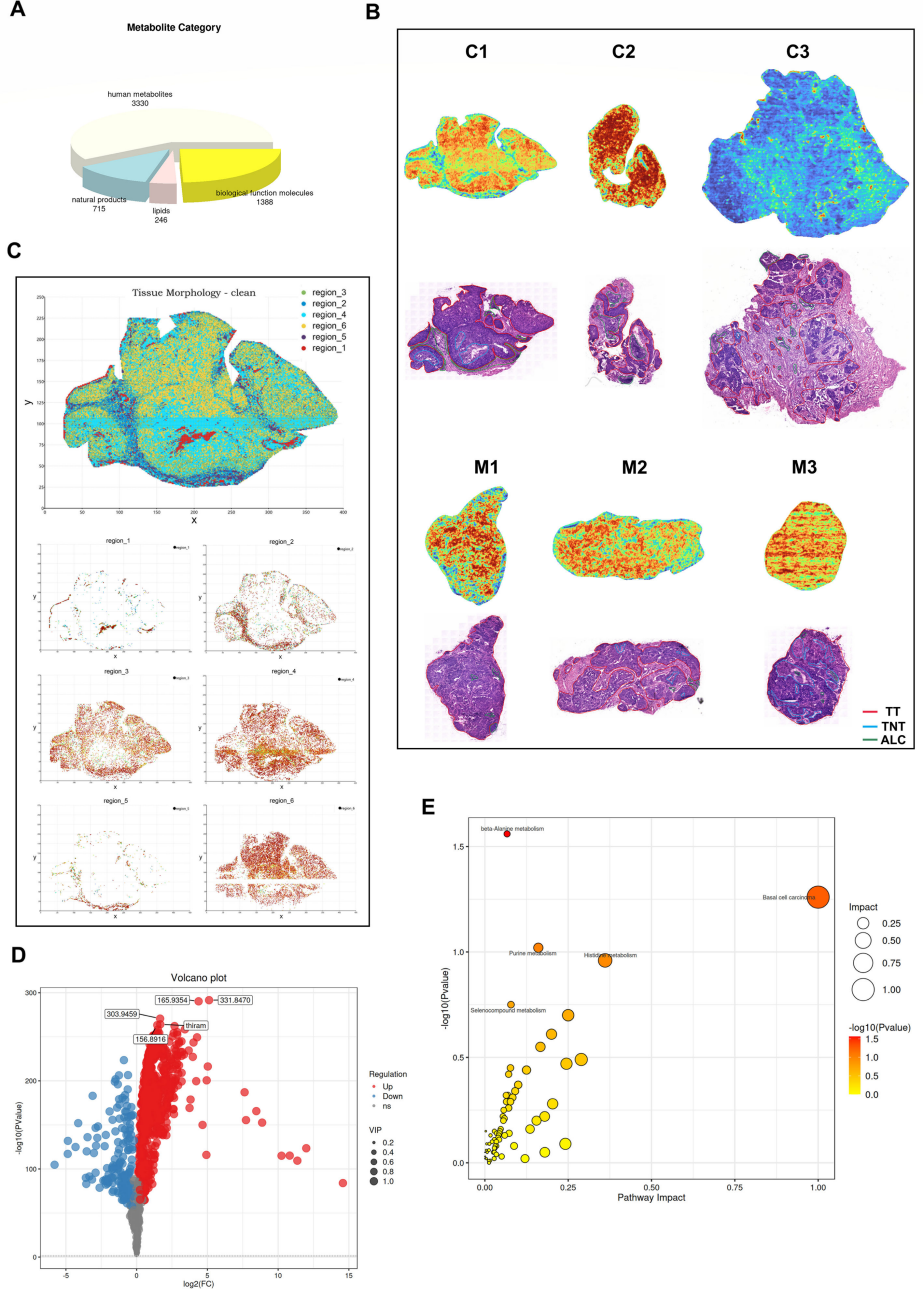
High-resolution spatial metabolomics characterize the metabolome in CC. (**A**) The pie chart showing the classification statistics of identified metabolites. (**B**) H&E stain images of different cervical cancer tissue regions (TT, tumor tissue; TNT, tumor necrotic tissue; and ALC, aggregated lymphocytes) from three CC tissue samples without LNM (**C**) and three CC tissue samples with LNM (M), and the spatial imaging distribution of total ion signals of metabolites in these tissue samples. Red represents a high metabolite total, and blue represents a low metabolite total. (**C**) Region-specific MS images of tumor tissue section from patient “No. C1.” (**D**) The volcano map showing the distribution and change trend of differential metabolites between CC with LNM and CC without LNM groups (M vs C). (**E**) The impact of differential metabolites on the enriched pathways by the KEGG pathway enrichment analysis.

To further explore the differences in the spatial distribution of the metabolome between CC with and without LNM, six surgical specimens from six individuals with CC (three with LNM and three without LNM) were sectioned into 10 μm frozen sections and subjected to high-resolution spatial metabolomics at a spatial resolution of 17 μm. A total of 3,330 metabolites were detected (Fig. 5A). Spatial imaging distribution analysis of the total ion signal of metabolites on the sample slices revealed intra- and inter-sample variations in the spatial distribution of relative metabolite abundance (Fig. 5B). Additionally, Fig. 5B presents H&E images of CC tissues from the six patients, indicating that the cancer samples primarily consist of tumor tissues, with a significant number of scattered and aggregated lymphocytes distributed in the interstitial tissues and noticeable necrotic areas in the central region of some tumor tissues. Tissue section segmentation maps were constructed based on the expression patterns and characteristics of metabolites, resulting in the division of six regions (Fig. 5C). For instance, in the CC tissue from patient No. C1, regions 1, 3, 4, and 6 represent a class of metabolites that are significantly upregulated in tumor tissues. Regions 2 and 5 characterize metabolites that are highly expressed in interstitial tissues, with region 2 specifically representing metabolites upregulated in aggregated lymphocytes within interstitial tissues (Fig. 5C). In addition, we plotted fold changes (using volcano plots) in the levels of metabolites identified through high-resolution spatial metabolomics in CC with LNM relative to CC without LNM, considering the statistically significant difference (*P* value) and variable importance in the projection. As shown in Fig. 5D, CC tissues with LNM demonstrated distinct metabolic profiles compared to those without LNM, with 642 metabolites upregulated and 151 metabolites downregulated. Consequently, high-resolution spatial metabolomics also revealed that alterations in tumor metabolism were involved in LNM of CC. Furthermore, consistent with the findings from global untargeted metabolomics, the metabolites exhibiting upregulated levels appear to be more prevalent in CC tissues with LNM. Detailed differential metabolite information for the two groups is provided in Table S4. Additionally, KEGG pathway enrichment analysis was conducted, indicating that several pathways were altered during LNM of CC, including the basal cell carcinoma pathway, histidine metabolism pathway, and pentose phosphate pathway, among others. However, with the exception of the β-alanine metabolism pathway, the alterations observed in the other pathways were not statistically significant (Fig. 5E).

### Spatial distribution of the differential metabolites detected by global untargeted metabolomics

**Fig 6:**
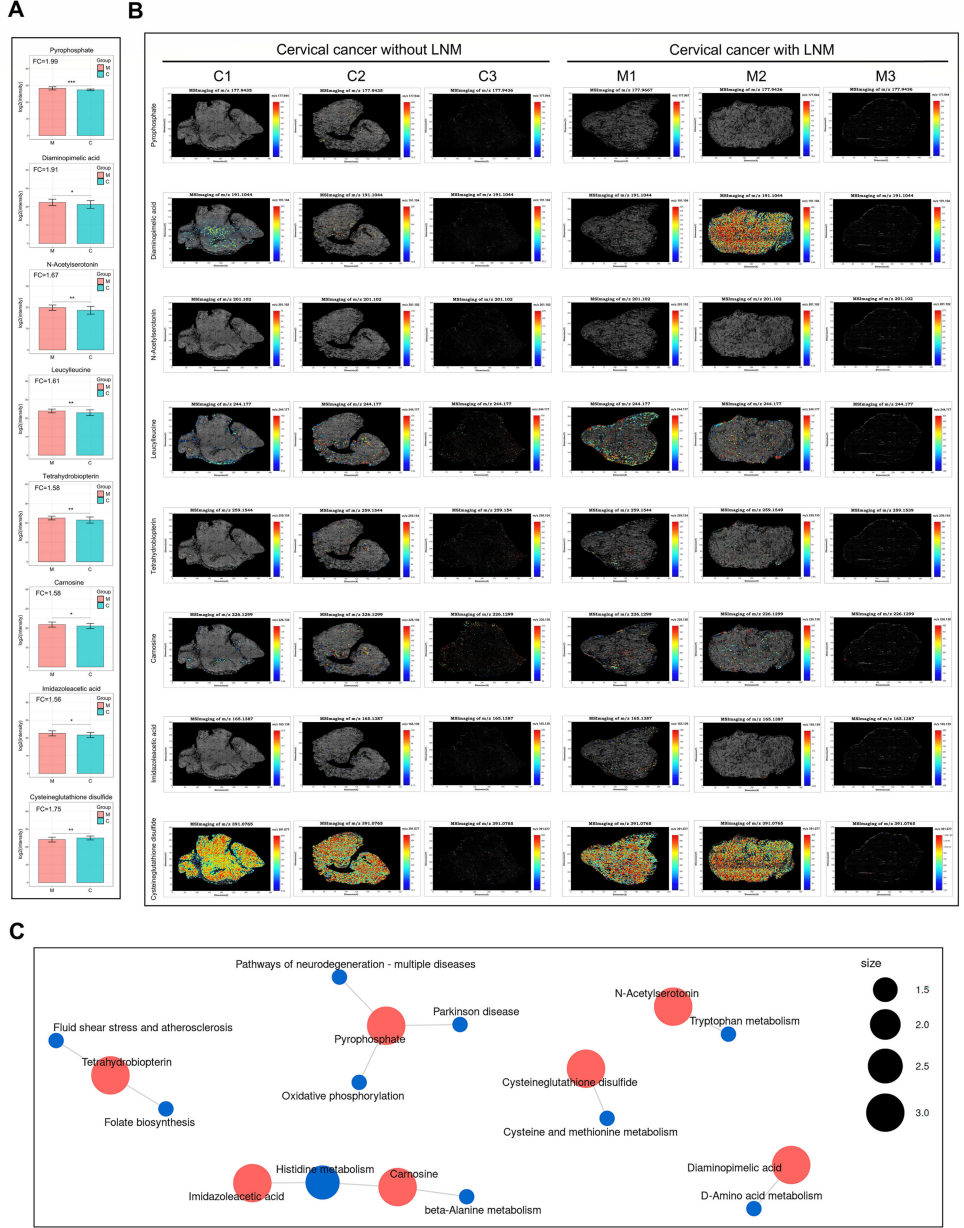
Spatial distribution of the differential metabolites detected by global untargeted metabolomics. (**A**) The expression levels of pyrophosphate, diaminopimelic acid, N-acetylserotonin, leucyl-leucine, tetrahydrobiopterin, carnosine, imidazoleacetic acid, and cysteine-glutathione disulfide in tissues of CC with (M, *n* = 3) and without LNM (C, *n* = 3) as measured by global untargeted metabolomics. (**B**) The MS images of pyrophosphate, diaminopimelic acid, N-acetylserotonin, leucyl-leucine, tetrahydrobiopterin, carnosine, imidazoleacetic acid, and cysteine-glutathione disulfide in tissues of CC with (M, *n* = 3) and without LNM (C, *n* = 3). (**C**) The pathways that pyrophosphate, diaminopimelic acid, N-acetylserotonin, leucyl-leucine, tetrahydrobiopterin, carnosine, imidazoleacetic acid, and cysteine-glutathione disulfide participate. Blue dots represent pathways, and red dots represent metabolites. The size of the pathway dots indicates the number of associated metabolites. Larger dots signify more metabolites.

To further elucidate the role of differential metabolites identified through global untargeted metabolomics in CC LNM, high-resolution spatial metabolomics analysis was conducted on six CC tissue samples. Among the 81 differential metabolites with a fold change ≥ 1.8 in the global untargeted metabolomics analysis, eight metabolites—pyrophosphate, diaminopimelic acid, N-acetylserotonin, leucyl-leucine, tetrahydrobiopterin, carnosine, imidazoleacetic acid, and cysteine-glutathione disulfide—were detected by high-resolution spatial metabolomics (Fig. 6A and B). As depicted in Fig. 6B, most of these metabolites are predominantly expressed in tumor tissues, with leucyl-leucine also exhibiting high expression in interstitial tissue. KEGG enrichment analysis revealed that these metabolites were primarily involved in amino acid metabolic pathways, in addition to oxidative phosphorylation, folate biosynthesis, and others (Fig. 6C). Notably, a greater number of metabolites were involved in the histidine metabolism pathway (Fig. 6C).

### Increased phospholipids are associated with LNM of CC

**Fig 7:**
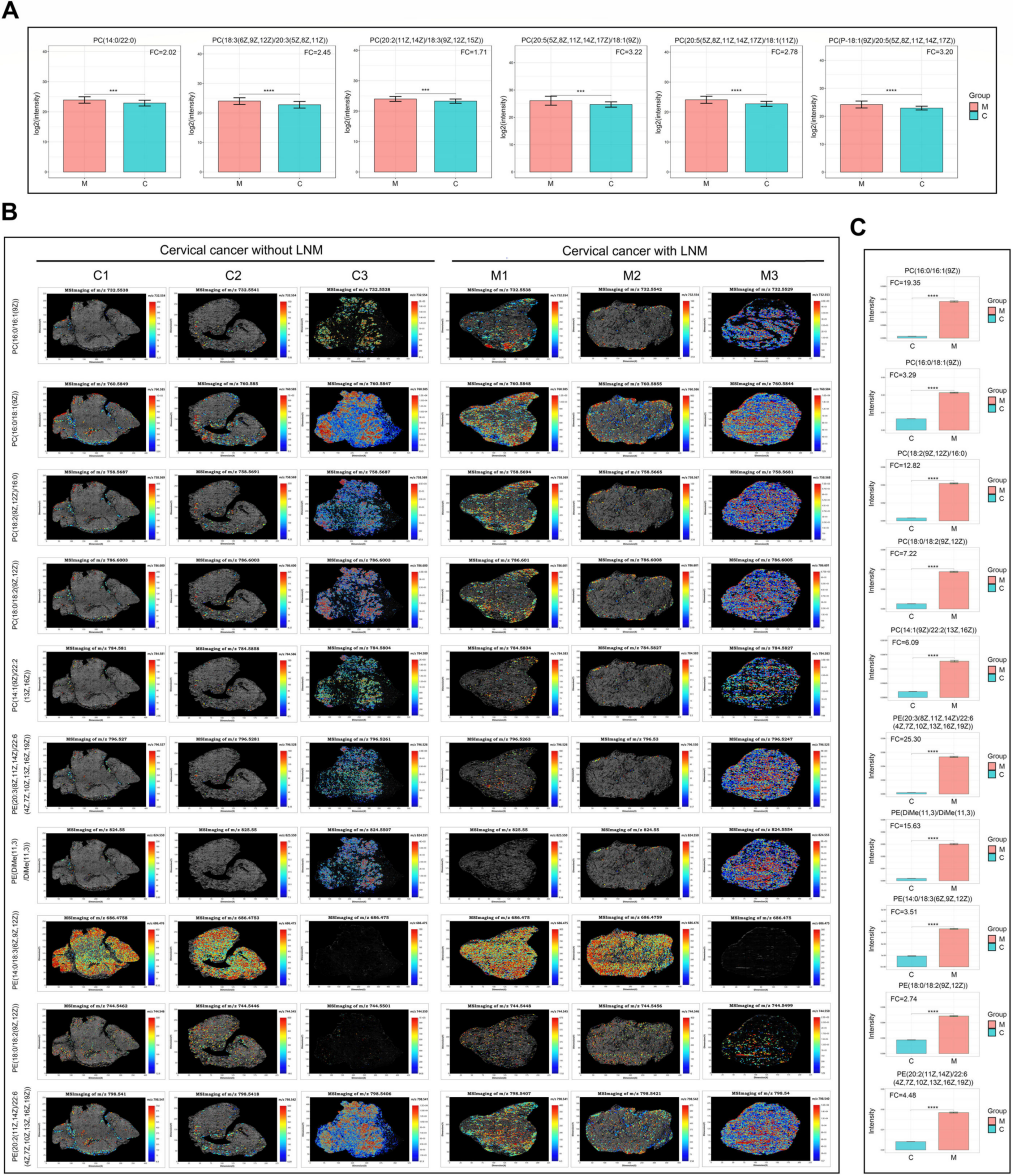
Increased phospholipids are associated with LNM of CC. (**A**) The expression levels of PC(14:0/22:0), PC[18:3(6Z,9Z,12Z)/20:3(5Z,8Z,11Z)], PC[20:2(11Z,14Z)/18:3(9Z,12Z,15Z)], PC[20:5(5Z,8Z,11Z,14Z,17Z)/18:1(9Z)], PC[20:5(5Z,8Z,11Z,14Z,17Z)/18:1(11Z)], and PC[P-18:1(9Z)/20:5(5Z,8Z,11Z,14Z,17Z)] in tissues of CC with (M, *n* = 25) and without LNM (C, *n* = 35) as measured by global untargeted metabolomics. (**B**) The MS images of PC[16:0/16:1(9Z)], PC[16:0/18:1(9Z)], PC[18:2(9Z,12Z)/16:0], PC[18:0/18:2(9Z,12Z)], PC[14:1(9Z)/22:2(13Z,16Z)], PE[20:3(8Z,11Z,14Z)/22:6(4Z,7Z,10Z,13Z,16Z,19Z)], PE[DiMe(11,3)/DiMe(11,3)], PE[14:0/18:3(6Z,9Z,12Z)], PE[18:0/18:2(9Z,12Z)], and PE[20:2(11Z,14Z)/22:6(4Z,7Z,10Z,13Z,16Z,19Z)] in tissues of CC (M, *n* = 3) with and without LNM (C, *n* = 3). (**C**) The expression levels of PC[16:0/16:1(9Z)], PC[16:0/18:1(9Z)], PC[18:2(9Z,12Z)/16:0], PC[18:0/18:2(9Z,12Z)], PC[14:1(9Z)/22:2(13Z,16Z)], PE[20:3(8Z,11Z,14Z)/22:6(4Z,7Z,10Z,13Z,16Z,19Z)], PE[DiMe(11,3)/DiMe(11,3)], PE[14:0/18:3(6Z,9Z,12Z)], PE[18:0/18:2(9Z,12Z)], and PE[20:2(11Z,14Z)/22:6(4Z,7Z,10Z,13Z,16Z,19Z)] in tissues of CC with (M, *n* = 3) and without LNM (C, *n* = 3) as measured by high-resolution spatial metabolomics.

In this study, the levels of phospholipids, particularly phosphatidylcholines (PCs) and phosphatidylethanolamines (PEs), were found to be elevated in CC with LNM compared to CC without LNM. Global untargeted metabolomics conducted on 60 CC tissue samples revealed significant increases in the levels of PC(14:0/22:0), PC[18:3(6Z,9Z,12Z)/20:3(5Z,8Z,11Z)], PC[20:2(11Z,14Z)/18:3(9Z,12Z,15Z)], PC[20:5(5Z,8Z,11Z,14Z,17Z)/18:1(9Z)], PC[20:5(5Z,8Z,11Z,14Z,17Z)/18:1(11Z)], and PC[P-18:1(9Z)/20:5(5Z,8Z,11Z,14Z,17Z)] in CC tissues with LNM (Fig. 7A). Additionally, high-resolution spatial metabolomics conducted on six CC tissue samples identified a significant upregulation of numerous phospholipids in CC tissues with LNM, including PC[16:0/16:1(9Z)], PC[16:0/18:1(9Z)], PC[18:2(9Z,12Z)/16:0], PC[18:0/18:2(9Z,12Z)], PC[14:1(9Z)/22:2(13Z,16Z)], PE[20:3(8Z,11Z,14Z)/22:6(4Z,7Z,10Z,13Z,16Z,19Z)], PE[DiMe(11,3)/DiMe(11,3)], PE[14:0/18:3(6Z,9Z,12Z)], PE[18:0/18:2(9Z,12Z)], and PE[20:2(11Z,14Z)/22:6(4Z,7Z,10Z,13Z,16Z,19Z)], with these phospholipids predominantly exhibiting stronger expressions in tumor tissues (Fig. 7B and C; Fig. S1A and B). The detailed information of the differential phospholipids revealed by high-resolution spatial metabolomics is presented in Table S4. Although the differential phospholipid molecules detected by global untargeted metabolomics and high-resolution spatial metabolomics differed, a positive association between phospholipid levels in tumor tissue and LNM of CC is evident. PC and PE are synthesized by combining fatty acids with phosphocholine and phosphoethanolamine under the catalysis of CHKA, ETNK, and CEPT1 and can be metabolized to fatty acids, choline, and ethanolamine through the catalysis of PLD and PLA2. IHC for CHKA, ETNK, CEPT1, PLD, and PLA2 was performed on the six surgical specimens used for high-resolution spatial metabolomics. The results suggested that CEPT1 and PLA2 expression was upregulated and downregulated, respectively, in CC with LNM compared to CC without LNM, implying their potential involvement in CC LNM through the regulation of PC and PE levels (Fig. S1).

## DISCUSSION

CC is one of the most prevalent malignant tumors in women worldwide (1). LNM is the most common and significant metastasis pathway of CC, serving as an independent risk factor affecting prognosis (2). Recent studies have highlighted the influence of intratumor microbiota and tumor metabolism on cancer progression. In this study, we initially characterized the intratumor microbiota and tumor metabolism associated with CC LNM, investigating the relationship between CC intratumor microbiota and tumor metabolism through the application of 16S rRNA sequencing, global untargeted metabolomics, and high-resolution spatial metabolomics on surgical CC specimens. Initially, we demonstrated the presence of tissue-resident bacteria in cervical cancer and paracancer tissues using IHC, aligning with previous research. Studies have suggested that intratumor microbes primarily originate from mucosal sites through mucosal barriers, adjacent normal tissues, and hematologic diffusion (12). Our findings revealed a higher abundance of bacteria in cervical paracancer tissues compared to CC tissues, supporting the notion that intratumor microbes may originate from adjacent normal tissues. Furthermore, we observed distinct compositions and diversities of intratumor microbiota between CC with LNM and CC without LNM. Each group exhibited specific bacterial genera that could potentially serve as diagnostic tools for CC LNM. In comparison to the CC without LNM group, the relative abundances of *Virgibacillus*, *Priestia*, *Anaerococcus*, *Sneathia, Campylobacter, Saccharopolyspora, Porphyromonas*, and *Peptostreptococcus* were significantly reduced in the CC with LNM group, suggesting their potential inhibitory role in CC LNM. *Anaerococcus* and *Porphyromonas* have been previously linked to prostate cancer metastasis, and *Porphyromonas gingivalis* has been shown to be positively associated with lymph node metastases in esophageal squamous cell carcinoma and oral squamous cell carcinoma patients (20–22). These findings appear contradictory to our results, suggesting a unique regulatory role for these two bacterial genera in CC LNM. Additionally, *Virgibacillus*, *Priestia*, *Anaerococcus*, and *Peptostreptococcus* all belong to the *Bacillota* phylum. Numerous studies have demonstrated a positive correlation between *Bacillota* and the occurrence of various tumors, such as breast cancer, esophageal adenocarcinoma, gastric cancer, and so on (23). However, its role in tumor metastasis remains unclear. Our study not only identified *Bacillota* as the dominant phylum in CC but also revealed that several bacterial genera within *Bacillota*, including *Virgibacillus*, *Priestia*, *Anaerococcus*, and *Peptostreptococcus*, exhibited a significant downward trend during CC LNM, suggesting their opposing role in CC occurrence and metastasis. Elevated levels of *Sneathia sanguinegens* have been associated with CC progression, and our study further demonstrated its negative effects on CC LNM (24). Moreover, the relative abundance of *Pseudomonadota* was found to be upregulated in the CC with LNM group, particularly *Caulobacter*, *Stenotrophomonas*, *Brucella*, *Hoeflea*, *Phenylobacterium*, and *Actinobacillus* within *Pseudomonadota*.

Studies have demonstrated that bacteria can participate in cancer cell invasion and metastasis by regulating cellular signaling pathways, gene expression, DNA damage, immune cell modulation, macrophage polarization, and host cell metabolism (23). Consequently, tumor metabolomics analysis of CC was conducted in our study. Correlation analysis between the tumor microbiome and metabolome revealed close associations between intratumor microbes and the levels of tetrahydrobiopterin, cysteine-glutathione disulfide, 15-demethoxy-epsilon-rhodomycin, 22-hydroxydocosanoic acid, and others. Tetrahydrobiopterin (BH_4_) is well established as a molecule involved in tumor progression, although its role in this process remains controversial. BH4, acting as either a pro- or anti-tumoral molecule, participates in various biological processes, including tumor microenvironmental reprogramming, cell growth, metabolism, and metastasis, contributing to or hindering cancer development (25). Our findings support the role of BH4 in inhibiting LNM in CC. Cysteine-glutathione disulfide, L-lysine, 4-(glutamylamino) butanoate, and S-(2-methylpropionyl)-dihydrolipoamide-E are all involved in amino acid metabolism (cysteine, methionine, lysine, arginine, proline, valine, leucine, and isoleucine). Increasing evidence indicates that amino acid metabolism is deregulated in numerous cancers and is involved in cancer progression in both tumorigenic and tumor-suppressive ways. Cysteine-glutathione disulfide, an oxidized form of glutathione, has been found to be negatively correlated with tumor progression (26). In our study, cysteine-glutathione disulfide was downregulated in the CC with LNM group, consistent with previous studies. However, Sun et al. (27) reported that in colorectal cancer, a lower glutathione to oxidized glutathione ratio was associated with liver metastases, seemingly contradicting our findings. These results suggest a dual role for cysteine-glutathione disulfide in different cancers. 4-(Glutamylamino) butanoate, a polyamine produced by the catabolism of arginine, may be involved in protein synthesis inhibition processes at high concentrations (28). Polyamines have been implicated in angiogenesis and metastasis, but their role in cancer promotion or inhibition remains controversial (29). In our study, the significant reduction of 4-(glutamylamino) butanoate in the CC with LNM group supports its potential inhibitory role in CC LNM. S-(2-methylpropionyl)-dihydrolipoamide-E, a product of branched-chain amino acid (BCAA) (valine, leucine, and isoleucine) catabolism, was shown to be significantly downregulated with CC LNM in our study, suggesting that low BCAA catabolism is associated with CC LNM. Several studies have implicated that low BCAA catabolism contributes to cancer progression, including hepatocellular carcinoma, breast cancer, leukemia, early pancreatic ductal adenocarcinoma, and clear cell renal cell carcinoma (30). The reduction of BCAA catabolism is often accompanied by elevated BCAA levels in tumor tissues. Increased BCAA levels can promote tumor progression through the activation of mTORC1 (30). Compared with CC without LNM, L-lysine was also found to be elevated in CC with LNM. Lysine, an essential amino acid in the human body, meets the high demand of tumor cells for amino acids, supporting tumor cell proliferation and cancer progression. Inosine, a nucleoside involved in purine production, gene translation, and controlling RNA destiny, has been reported to enhance the anti-tumor immune response through various mechanisms (31). In this study, we observed a significant downregulation of inosine with CC LNM. Therefore, we speculate that the loss of anti-tumor immune response due to decreased tumor inosine levels may be one of the factors contributing to LNM in CC.

Lipid metabolic reprogramming is an emerging hallmark of cancer (32). Increased lipid uptake, storage, and lipogenesis occur in various cancers and contribute to cancer progression. Phospholipids, including PC, PE, phosphatidylserine, phosphatidylinositol (PI), and phosphatidic acid, are the major constituents of biological membranes. Growing evidence demonstrates that phospholipid synthesis is enhanced in cancer, regulating tumor metastasis by modulating changes in membrane phospholipid composition and producing bioactive lipid second messengers (33). The phospholipid composition and degree of saturation determine the biophysical properties of membranes and influence various cellular processes. Previous studies have indicated that elevated levels of unsaturated phospholipids are associated with increased membrane fluidity, thereby facilitating tumor metastasis (34, 35). In this study, we identified PE and PC as the most significantly altered phospholipids. Notably, we observed a substantial increase in unsaturated PCs and PEs, particularly polyunsaturated species such as PCs (18:2/16:0, 18:0/18:2, 14:1/22:2, 18:3/20:3, P-18:1/20:5, 20:5/18:1) and PEs (20:0/20:3, 20:3/22:6, 20:2/22:6, 14:0/18:3, 18:0/18:2) in CC tissues with LNM. These findings appear to be consistent with the conclusions of previous studies. Lipid droplet (LD) formation is a prevalent phenotype observed across various cancer types, with a number of mechanisms that are involved in supporting metastasis (36). The phospholipid membrane of LDs mainly consists of PC, comprising up to 60% of the total phospholipid content, followed by PE (24%) and PI (8%) (37). Under conditions of very active lipid droplet expansion, the synthesis of new phospholipids is essential for maintaining phospholipid homeostasis (38). Consequently, we propose that the upregulation of PCs and PEs may facilitate LNM in CC by enhancing lipid droplet formation. Moreover, PEs have been shown to positively regulate cell division, ATP production, and longevity in mammalian cells, all of which contribute to tumor progression (39). Further mechanistic exploration revealed that CEPT1, a molecule responsible for catalyzing PC and PE synthesis, appeared to be upregulated in CC tissues with LNM, suggesting that increased PC and PE synthesis may be involved in CC LNM. Additionally, the relative abundance of intratumor microbes decreased significantly with CC LNM, all seemingly negatively correlated with the level of tumor phospholipids, although the correlation was not statistically significant. Collectively, these findings suggest that the increased tumor phospholipid levels caused by CC intratumor microbes may be another contributing factor to CC LNM. We believe that this speculation could be confirmed by increasing the sample size.

Despite the valuable contributions of our study, several limitations remain. Our study utilized observational methods to characterize the alterations in tumor microbiome and metabolome associated with CC LNM, but it did not establish a causal relationship between these alterations and CC LNM. The absence of an external validation cohort and a power analysis to justify the sample size limited the robustness of the findings. Additionally, some factors that influence the composition of intratumor microbiota, such as socio-economic factors and diet, were not considered due to specific objective constraints. Consequently, to elucidate the roles of the altered microbes and metabolites in CC LNM, further comprehensive, systematic, and in-depth investigations are needed.

In summary, our study comprehensively characterized the microbiome and metabolome of CC with and without LNM through the application of 16S rRNA sequencing, global untargeted metabolomics, and high-resolution spatial metabolomics. Our findings identified multiple intratumor microbes and tumor metabolites associated with CC LNM and preliminarily described the potential relationships between these intratumor microbes and tumor metabolites. These results not only offer potential microbial and metabolic diagnostic biomarkers and therapeutic targets for CC LNM but also contribute to a deeper understanding of the underlying molecular mechanisms of CC LNM.

## Data Availability

Key raw data of 16S rRNA sequencing are available in the National Center for Biotechnology Information Sequence Read Archive (BioProject ID: PRJNA1289904). Global untargeted metabolomics data are available in the Open Archive for Miscellaneous Data of the National Genomics Data Center (OMIX ID: OMIX010944). Other datasetsdata sets used and/or analysedanalyzed during the current study are available from the corresponding author on reasonable request.

## SUPPLEMENTAL MATERIAL

Supplemental figures spectrum.02247-25-s0001.docx Fig. S1 to S3.

Supplemental material spectrum.02247-25-s0002.docx Supplemental methods.

Table S1 spectrum.02247-25-s0003.xlsx The LEfSe analysis results for CC with and without LNM.

Table S2 spectrum.02247-25-s0004.xlsx The differential metabolites between the CC with and without LNM groups identified through global non-targeted metabolomics.

Table S3 spectrum.02247-25-s0005.xlsx The results of the KEGG enrichment analysis for the differential metabolites identified through global non-targeted metabolomics.

Table S4 spectrum.02247-25-s0006.xlsx The differential metabolites between the CC with and without LNM groups identified through high-resolution spatial metabolomics.

ASM does not own the copyrights to Supplemental Material that may be linked to, or accessed through, an article. The authors have granted ASM a non-exclusive, world-wide license to publish the Supplemental Material files. Please contact the corresponding author directly for reuse.
