# Supplementary material for: Multi-omics analysis reveals the alterations in the tumor microbiome and metabolome associated with cervical cancer lymph node metastasis
Source: Microbiol Spectr. 2026 Apr 30;14(6):e02247-25. doi: 10.1128/spectrum.02247-25 (PMC13228043; doi:10.1128/spectrum.02247-25)
Supplement: Supplemental figures — Fig. S1 to S3. [file spectrum.02247-25-s0001.docx]

**
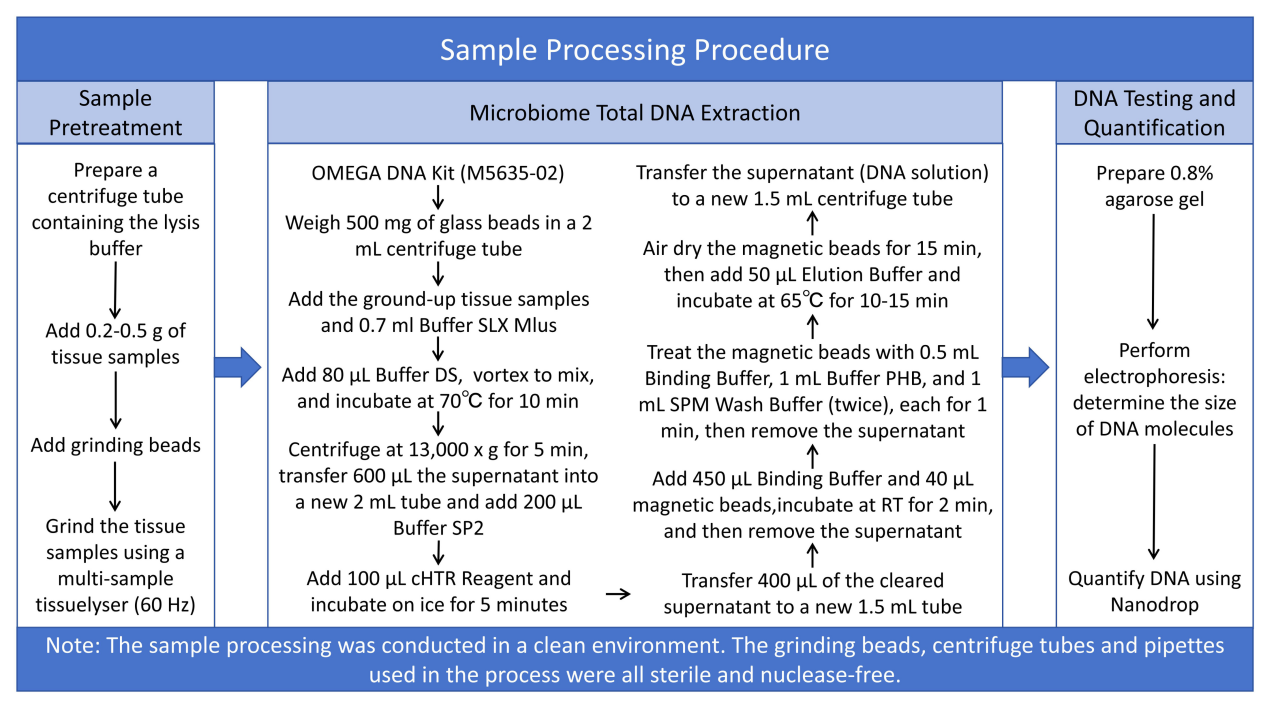
Figure S1. The sample preparation process for 16S rRNA gene amplicon sequencing**

**

Figure S2. Region-specific expression of representative phospholipids in tissues of CC with and without LNM**

1. Region-specific MS images of tumor tissue sections from patients with and without LNM.
2. The expression levels of PC(16:0/18:1(9Z)), PC(18:2(9Z,12Z)/16:0), PC(18:0/18:2(9Z,12Z)), PE(20:3(8Z,11Z,14Z)/22:6(4Z,7Z,10Z,13Z,16Z,19Z)), PE(18:0/18:2(9Z,12Z)) and PE(20:2(11Z,14Z)/22:6(4Z,7Z,10Z,13Z,16Z,19Z)) in different tissue regions of CC.


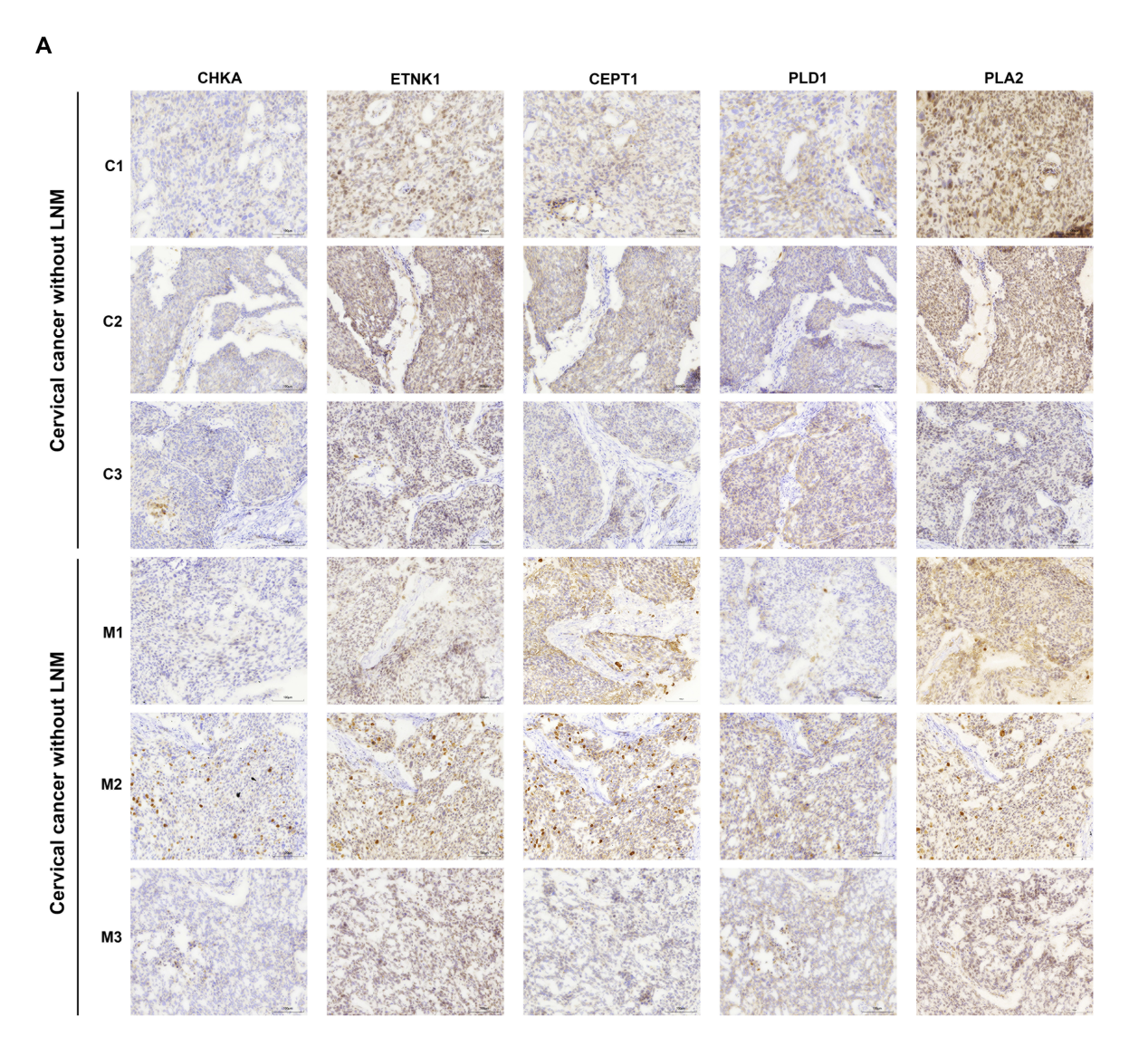


**Figure S3. The expression of the molecules that catalyze the synthesis and metabolism of PC and PE in CC with and without LNM**

1. Immunohistochemical staining for CHKA, ETNK, CEPT1, PLD and PLA2 using the tumor tissue section from CC patients with (M) and without LNM (C).
