# Supplementary material for: Multi-omics analysis reveals the alterations in the tumor microbiome and metabolome associated with cervical cancer lymph node metastasis
Source: Microbiol Spectr. 2026 Apr 30;14(6):e02247-25. doi: 10.1128/spectrum.02247-25 (PMC13228043; doi:10.1128/spectrum.02247-25)
Supplement: Supplemental material — Supplemental methods. [file spectrum.02247-25-s0002.docx]

**Metabolite extraction and global untargeted metabolomic analysis**

**Metabolite extraction**

An appropriate amount of sample was weighed accurately into a 2 mL centrifuge tube, added 1000 µL tissue extract【75 %（9:1 methanol: chloroform):25% H2O】, and steel balls. The centrifuge tube was put into the tissue grinder, and ground at 50 Hz for 60 s. The above operation was repeated twice. The sample was ultrasounded for 30 min at room temperature, and bathed in ice for 30 minutes. The sample was centrifuged for 10 min at 12,000 rpm and 4℃. The supernatant was transfered to a new 2 mL centrifuge tube, concentrated and dried. 200 µL 2-chloro-l-phenylalanine (4 ppm) solution with 50% acetonitrile solution was added to redissolve the sample. The supernatant was filtered by 0.22 μm membrane, and transferred into the detection bottle for LC-MS detection.

**Liquid chromatography conditions**

The LC analysis was performed on a Vanquish UHPLC System (Thermo Fisher Scientific, USA). Chromatography was carried out with an ACQUITY UPLC ® HSS T3 (2.1×100 mm, 1.8 µm) (Waters, Milford, MA, USA). The column maintained at 40 ℃. The flow rate and injection volume were set at 0.3 mL/min and 2 μL, respectively. For LC-ESI (+)-MS analysis, the mobile phases consisted of 0.1% formic acid in acetonitrile (v/v) (B2) and 0.1% formic acid in water (v/v) (A2). Separation was conducted under the following gradient: 0~1 min, 8% B2；1~8 min, 8%~98% B2; 8~10 min, 98% B2; 10~10.1 min, 98%~8% B2; 10.1~12 min, 8% B2. For LC-ESI (-)-MS analysis, the analytes was carried out with acetonitrile (B3) and ammonium formate (5mM) (A3). Separation was conducted under the following gradient: 0~1 min, 8% B3; 1~8 min; 8%~98% B3; 8~10 min, 98% B3; 10~10.1 min, 98%~8% B3; 10.1~12 min, 8% B3.

**Mass spectrum conditions**

Mass spectrometric detection of metabolites was performed on Orbitrap Exploris 120 (Thermo Fisher Scientific, USA) with ESI ion source. Simultaneous MS1 and MS/MS (Full MS-ddMS2 mode, data-dependent MS/MS) acquisition was used. The parameters were as follows: sheath gas pressure, 40 arb; aux gas flow, 10 arb; spray voltage, 3.50 kV and -2.50 kV for ESI(+) and ESI(-), respectively; capillary temperature, 325 ℃; MS1 range, m/z 100-1000; MS1 resolving power, 60000 FWHM; number of data dependant scans per cycle, 4; MS/MS resolving power, 15000 FWHM; normalized collision energy, 30%; dynamic exclusion time, automatic.

**Data preprocessing**

The raw data were firstly converted to mzXML format by MSConvert in ProteoWizard software package (v3.0.8789) and processed using R XCMS (v3.12.0) for feature detection, retention time correction and alignment. Key parameters settings were set as follows: ppm = 15, peakwidth = c (5, 30), mzdiff = 0.01, method = centWave. The batch effect was then eliminated by correcting the data based on QC samples. Metabolites with RSD > 30% in QC samples were filtered and then used for subsequent data analysis. The metabolites were identified by accuracy mass and MS/MS data which were matched with HMDB (http://www.hmdb.ca), massbank (http://www.massbank.jp/), KEGG (https://www.genome.jp/kegg/), LipidMaps (http://www.lipidmaps.org), mzcloud (https://www.mzcloud.org) and the metabolite database bulid by Panomix Biomedical Tech Co., Ltd. (Suzhou, China). The molecular weight of metabolites was determined according to the m/z (mass-to-charge ratio) of parent ions in MS data. Molecular formula was predicted by ppm (parts per million) and adduct ion, and then matched with the database. At the same time, the MS/MS data from quantitative table of MS/MS data, were matched with the fragment ions and other information of each metabolite in the database, so as to realize the MS/MS identification of metabolites.

**Data analysis**

Two different multivariate statistical analysis models, unsupervised and supervised, were applied to discriminate the groups (PCA; PLS-DA; OPLS-DA) by R ropls (v1.22.0) package. The statistical significance of P.value was obtained by statistical test between groups. Finally, combined with P.value, VIP (OPLS-DA variable projection importance) and FC (multiple of difference between groups) to screen biomarker metabolites. By default, when P value < 0.05 and VIP value > 1, we think that metabolite were considered to have significant differential expression.

**Pathway analysis**

Differential metabolites were subjected to pathway analysis by MetaboAnalyst, which combines results from powerful pathway enrichment analysis with the pathway topology analysis. The identified metabolites in metabolomics were then mapped to the KEGG pathway for biological interpretation of higher-level systemic functions. The metabolites and corresponding pathways were visualized using KEGG Mapper tool.

**Spatial** **metabolomics analysis**

**Preparation of frozen section**

Using a cryostat (Thermo CRYOSTAR NX50), tissues were put into the -20 °C freezer for 3 hours to equilibrate, and then sectioned at 10 μm thickness for mass spectrometry imaging and HE staining. Transfer the sections to a pre-cooled ITO conductive glass slide with a pre-cooled brush and dry for 20 min. If the sliced samples are not tested immediately, the ITO conductive glass slides can be stored at -80 °C.

**HE staining**

The sliced samples were baked in an oven at 60°C for 25 minutes, and rinsed with slow water flow for 1~3 seconds. Hematoxylin staining was conducted for 2-3 minutes, and rinsed with running water for 5~10 seconds. The slides were soaked in 75% ethanol containing 1% HCl for 20-30 seconds; PBS solution for 30 seconds, PBS solution for 30 seconds, and 95% ethanol for 4-5 minutes (repeated twice) in turn. The slides were dried and added the mounting fluid dropwise according to the size of the samples, and were covered with a cover slip.

**Matrix spray**

Desiccated tissue sections were sprayed with 10 mg/mL α-CHCA (α-cyano-4-hydroxycinnamic acid) dissolved in 90 %:10 % Methanol: Trifluoroacetic acid using a robotic aerosol sprayer (SunCollect, SunChrom). The nozzle temperature was set to room temperature with a moving velocity of 1000 mm/min. Sixteen passes of matrix were deposited at a gradient flow rate ranging from 10-60 μL/min.

**Mass spectrum conditions**

An AP/MALDI (ng) UHR ion source (MassTech, Columbia, MD) was coupled with Thermo QE Plus mass spectrometer (Thermo Fisher Scientific, USA) for all data acquisition. The laser energy of 15% was used. The mass spectrometer was operated at full MS mode with positive polarity. Capillary temperature was set to 320℃and S-lens RF level was 50%. During full scans, mass range of m/z 120-1000 was used with resolution of 35,000. Automatic gain control (AGC T) target of 1e6 with 100 ms maximum injection time was used. An isolation window of m/z 2 was used.
